# Effect of behavioural activation for individuals with post-stroke depression: systematic review and meta-analysis

**DOI:** 10.1192/bjo.2024.721

**Published:** 2024-07-30

**Authors:** Engida Yisma, Sandra Walsh, Susan Hillier, Marianne Gillam, Richard Gray, Martin Jones

**Affiliations:** Department of Rural Health, University of South Australia, Allied Health & Human Performance, Australia; and IIMPACT in Health, University of South Australia, Australia; IIMPACT in Health, University of South Australia, Australia; Department of Rural Health, University of South Australia, Allied Health & Human Performance, Australia; and School of Nursing and Midwifery, La Trobe University, Australia

**Keywords:** Behavioural activation, post-stroke depression, efficacy, systematic review, meta-analysis

## Abstract

**Background:**

Previous research showed that behavioural activation is as effective as cognitive–behavioural therapy for general depression. However, it remains unclear if it leads to greater improvement in depressive symptoms when compared with standard treatment for post-stroke depression.

**Aims:**

To compare the effectiveness of behavioural activation against control conditions in reducing depression symptoms in individuals with post-stroke depression.

**Method:**

This review searched five databases from inception until 13 July 2021 (updated 15 September 2023) for randomised controlled trials comparing behavioural activation and any control conditions for post-stroke depression. Risk of bias was assessed with the Cochrane Collaboration's Risk-of-Bias 2 tool. The primary outcome was improvement in depressive symptoms in individuals with post-stroke depression. We calculated a random-effects, inverse variance weighting meta-analysis.

**Results:**

Of 922 initial studies, five randomised controlled trials with 425 participants met the inclusion criteria. Meta-analysis showed that behavioural activation was associated with reduced depressive symptoms in individuals with post-stroke depression at 6-month follow-up (Hedges’ *g* −0.39; 95% CI −0.64 to −0.14). The risk of bias was low for two (40%) of five trials, and the remaining three (60%) trials were rated as having a high risk of bias. Heterogeneity was low, with no indication of inconsistency.

**Conclusions:**

Evidence from this review was too little to confirm the effectiveness of behavioural activation as a useful treatment for post-stroke depression when compared with control conditions. Further high-quality studies are needed to conclusively establish the efficacy of behavioural activation as a treatment option for post-stroke depression.

## Post-stroke depression

Post-stroke depression affects approximately a third of stroke survivors at some point after stroke.^1^ A 2023 systematic review and meta-analysis by Liu et al,^2^ involving 77 observational studies, found that the overall pooled prevalence of post-stroke depression was 27% at any time point after stroke, with a prevalence of 24% based on clinical interviews (clinician-rated) and 29% based on rating scales (self-reported). Post-stroke depression negatively affects stroke recovery, contributing to higher mortality and hospital readmission rates, reduced quality of life^3^ and decreased engagement in rehabilitation programmes.^4^ Caregivers are also affected by living with someone with post-stroke depression, because of constant exposure to the individual's struggles and the potential strain on relationships.^5^ Although many stroke survivors with post-stroke depression are prescribed antidepressants, these medications seem less effective for post-stroke depression than for depression not related to stroke.^1^ This reduced antidepressant efficacy may arise because stroke causes neurological changes that modify the underlying biology and drug responsiveness.^6^ Moreover, antidepressants have side-effects that may hinder stroke recovery.^7^ However, it has been reported that most people prefer psychological treatment over pharmacological treatment, because of a fear of side-effects/addictions.^8^ The National Institute for Health and Care Excellence clinical guidelines recommend a high-intensity psychological intervention, such as cognitive–behavioural therapy (CBT) for patients with moderate depression and a chronic physical health problem.^9^ Evidence from a 2018 meta-analysis of 23 randomised controlled trials (RCTs) involving 1972 participants found that CBT for post-stroke depression was associated with positive effect in alleviating the symptoms of depression.^10^ However, CBT is complex and costly.^11^ As the ‘cognitive’ component of CBT focuses on teaching skills for challenging negative thoughts, CBT involves a lengthy period of training of the therapist,^12^ and requires specialist qualifications as mental health workers to deliver the therapy.

## Behavioural activation as a candidate treatment for post-stroke depression

Behavioural activation, a component of CBT, has been used for decades as the ‘behavioural’ component of CBT or as stand-alone treatment for depression.^13^ The aim of behavioural activation is to reverse the cycle of depression by monitoring mood and increasing engagement in valued activities.^14^ Behavioural activation is easy to deliver and could be a candidate psychological intervention for individuals with post-stroke depression. It supports the person to engage in meaningful activities, and teaches skills to notice changes in mood and its relationship with these activities. Mastery of these activities provides fulfilment and reward. The aim of therapy is to help the patient schedule activity that is inherently rewarding. This engagement with rewarding activity may be particularly important for people with stroke who experience diminished physical capability. Training in behavioural activation usually takes about 5 days,^15^ and can be delivered by non-specialist mental health professionals.^16^ Behavioural activation is usually delivered face to face over six to ten sessions.^16,17^

A 2016 RCT by Richards et al^16^ examined the clinical efficacy and cost-effectiveness of behavioural activation compared with CBT for adults with depression. They found that behavioural activation is as effective as CBT, and can be delivered by junior mental health workers with less intensive and costly training. Moreover, a 2020 systematic review by Uphoff et al^18^ examined behavioural activation compared with other psychological therapies, medication, or treatment as usual/waiting list/placebo for depression in adults. They concluded that behavioural activation ‘may be more effective than humanistic therapy, medication, and treatment as usual, and that it may be no less effective than CBT, psychodynamic therapy, or being placed on a waiting list’.^18^ This means that modifying behaviour may be enough to improve depression, and it may be unnecessary to directly challenge negative thinking through CBT.

Moreover, behavioural activation could be suitable for post-stroke depression as its aim is to introduce behaviour that promotes mastery, pleasure and routine tailored to the individual.^19,20^ For many reasons, including apprehension, fear or avoidance, post-stroke survivors may disengage from activities that were once pleasurable. For example, a person may stop cooking post-stroke, an activity they previously found pleasurable, because of concerns of personal safety. Currently, there is limited evidence to confirm or refute whether behavioural activation would be beneficial for people with post-stroke depression. Therefore, we conducted this systematic review and meta-analysis to understand the effectiveness of behavioural activation as a psychological treatment for post-stroke depression.

## Method

We conducted a systematic review and meta-analysis of RCTs reporting on the effectiveness of behavioural activation on post-stroke depression. The procedures for the review were prespecified in a registered protocol (Open Science Framework: https://osf.io/7kqu3), and a statistical analysis plan was finalised before any analyses. This review relied on previously published material and did not require ethical approval. We followed the 2020 Preferred Reporting Items for Systematic Reviews and Meta-Analyses (PRISMA) reporting guidelines for systematic reviews and meta-analyses (Supplementary Appendix 1 available at https://doi.org/10.1192/bjo.2024.721).^21^

### Eligibility criteria

RCTs involving participants aged 18 years and older with post-stroke depression and who were treated with behavioural activation were eligible. We focused on studies involving behavioural activation as the primary treatment based on any type of delivery mode, including face-to-face or online, individual or group sessions. Peer-reviewed publications were reviewed and only studies published in English were included. Studies were not excluded based on sample size, follow-up period or year of publication.

### Participants/population

We included RCTs with adult participants over 18 years old, of any gender. Participants must have had depression (mild, moderate or severe) following a stroke.

### Intervention

The intervention of interest in this systematic review was behavioural activation. We included RCTs that assessed treatment approaches for post-stroke depression explicitly labelled as ‘behavioural activation’. Additionally, we considered trials that described the interventions utilising the core components of behavioural activation for depression, such as mood monitoring and activity scheduling. In cases where it was difficult to ascertain how the intervention was defined, we contacted the authors for clarification.

### Comparator

All comparators were considered acceptable if they did not fall under the category of behavioural activation. These included treatment as usual, comparative depression treatments, treatment as usual supplemented with antidepressants, or medical placebo.

### Outcomes

The main outcome measure was treatment efficacy for post-stroke depression. This was determined by examining changes in depression symptoms among stroke survivors from baseline to each follow-up point, as evaluated by standardised depression scales, such as the Hospital Anxiety and Depression Scale^22^ or the Patient Health Questionnaire-9 (PHQ-9).^23^ When a study included multiple instruments for the same outcome, only one scale was chosen, based on the most commonly used scale.^24^

### Search strategy

We systematically searched five databases (Medline, EMBASE, EMCARE, Cochrane Library and PsycINFO) from database inception up to 13 July 2021 (updated 15 September 2023). Hand-searching by examining reference lists of included studies and relevant reviews was also conducted to identify any further studies that could be included. The population search terms related to stroke included ‘stroke’, ‘poststroke’, ‘cerebrovascular’ and ‘cerebrovascular accident’. The population search terms related to depression included ‘depression’, ‘depressive’, ‘emotional depression’, ‘depressive symptom’, ‘mood’, ‘low mood’, ‘depressed’, ‘dysthymia’, ‘vascular depression’, ‘poststroke depression’ and ‘depressive disorder’. The intervention search terms (keywords) related to behavioural activation included ‘behavior* activation’, ‘behavior* therapy’, ‘activity schedul*’, ‘positive reinforce*’, ‘event schedul*’, ‘mood monitoring’, ‘behavio* treatment’, ‘behavio* intervention’, ‘behavio* modif*’ and ‘behavio* psychotherap*’. Full details of each search strategy are available in Supplementary Appendix 2 (Supplementary Tables 1–5).

### Study selection

Three review authors (E.Y., S.W. and M.J.) independently screened the titles and/or abstracts of all publications obtained through the search strategy. We then obtained full articles for all trials, and the same three review authors (E.Y., S.W. and M.J.) assessed the full texts according to criteria relating to study, participant, intervention and outcome characteristics. We discussed any disagreements with a third review author (of E.Y., S.W., M.J. or R.G.) to reach consensus. We recorded the reasons for excluding studies that did not meet the inclusion criteria. We constructed a PRISMA flow diagram to illustrate the study selection process.

### Data extraction

We used data extraction forms to retrieve information from the studies incorporated in this review. The data were extracted on 24 February 2022 (for the updated search, the data extraction was completed on 6 December 2023). Three authors (E.Y., S.W. and M.J.) independently extracted data from each trial. Any discrepancies among these authors were resolved through discussion with an additional member of the review team (chosen from E.Y., S.W. and M.J.). The information extracted from each trial included: (a) basic details, such as authors’ names, publication year, study design, follow-up duration, outcome measures (type and time points) and full intervention specifics (type, frequency, etc.); and (b) statistical data (mean and s.d.) for the primary outcome (post-stroke depression). To categorise treatment time points for post-treatment outcomes as well as outcomes at each reported follow-up point, we utilised the cut-offs described by Uphoff et al,^18^ defining short term as up to 6 months post-treatment, medium term as 7–12 months post-treatment and long term as more than 12 months post-treatment.

### Assessment of risk of bias in included studies

Two review authors (E.Y. and M.J.) assessed the risk of bias in included trials and discussed any disagreements with a third review author (R.G.). The risk-of-bias data from the included studies was summarised visually in graphs and is described narratively in the text. To evaluate the risk of bias in each of the trials included in the review, we used the Cochrane Collaboration's Risk of Bias Assessment Tool (version 2).^25^ The tool considers the following domains: (a) risk of bias arising from the randomisation process, including allocation and randomisation; (b) risk of bias due to deviations from the intended interventions, including blinding of participants and people delivering the interventions; (c) risk of bias due to missing outcome data; (d) risk of bias in measurement of the outcome, including blinding of outcome assessors and (e) risk of bias in the selection of reported results.

### Data synthesis and analysis

We conducted both a narrative synthesis and a meta-analysis of the findings from the included studies. The narrative synthesis summarised the results of each individual study in words and text. This allowed examination of the study characteristics, contexts and specific details that a meta-analysis cannot capture. The meta-analysis provided a quantitative pooling of the data to estimate the overall effect size of behavioural activation compared with control conditions (treatment as usual or usual care plus antidepressants) on reducing depression symptoms in individuals with post-stroke depression.

For each comparison between behavioural activation and a control condition, we calculated effect sizes as Hedges’ *g*, indicating the difference between the two groups at each follow-up point. As one study^26^ did not report means and s.d., we calculated the effect size by using the reported dichotomous outcome data.

We used a random-effects model for the meta-analysis, to account for expected heterogeneity across studies. Inverse variance weighting was used to pool effect sizes across the studies.

The meta-analysis synthesised data on short-term, medium-term and long-term efficacy of behavioural activation compared with control conditions. The summary effect size was reported as a Hedges’ *g* with a 95% confidence interval. Heterogeneity was assessed by examining the between-study heterogeneity parameter, τ. This parameter assumes that the variance between studies is consistent across all treatment contrasts, allowing the model to effectively capture between-study variability and improve the estimation of heterogeneity. All statistical analyses for the meta-analysis were performed with Stata/SE for Windows version 18.0 (Stata Corp., College Station, Texas, USA).

### Patient and public involvement

Patients and the public were not involved in this study, as it was to establish what had been done in the field. The next phase will involve patients in the research.

## Results

### Results of the search

**Fig. 1:**
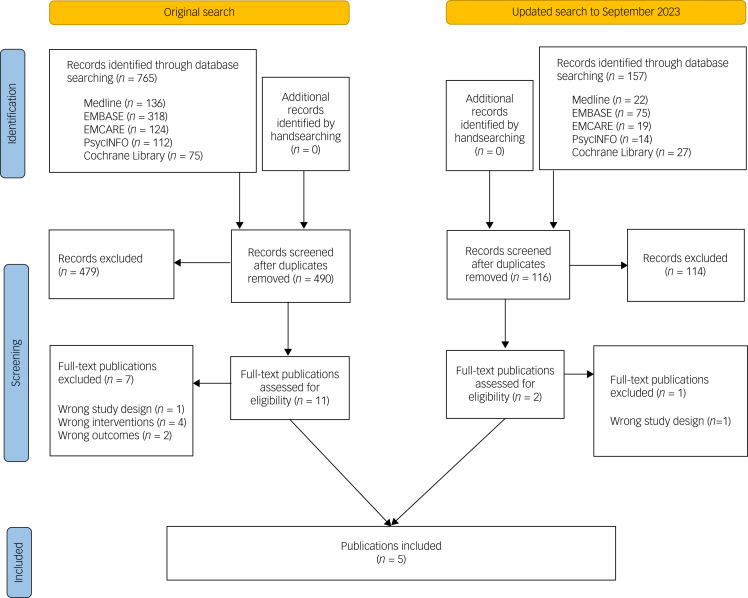
Flow of publications through different stages of the systematic review.

The flow of publications during the review process is shown in Fig. 1. The initial search identified 922 citations, of which 316 were duplicates and removed. A total of 593 citations were excluded during the title and abstract screening. We reviewed 13 full-text articles, but five studies were eligible to be included in the final review. The list of excluded full-text studies is provided in Supplementary Appendix 3.

### Description of studies

#### Included studies

The five included RCTs evaluated the effectiveness of behavioural activation for individuals with post-stroke depression. Together, these studies included 425 participants. We contacted authors for information needed to ascertain how the authors defined behavioural activation in their studies. The characteristics of studies included in this review are provided in Table 1.

**Table 1:** Characteristics of included studies

	Thomas et al, 2013^27^	Thomas et al, 2019^28^	Sun et al, 2022^29^	Mitchell et al, 2009^30^	Kirkness et al, 2017^26^
Country	UK	UK	China	USA	USA
Study design	RCT	RCT	RCT	RCT	RCT
Aim	To evaluate behavioural therapy as a treatment for low mood in people with aphasia	To assess the feasibility of conducting a definitive RCT of behavioural activation for treating post-stroke depression	To evaluate the feasibility and effectiveness of behavioural activation for subthreshold depression after stroke	To evaluate a brief psychosocial behavioural intervention plus antidepressant for post-stroke depression	To compare a shortened psychosocial behavioural intervention delivered by telephone or in person with usual care for treating post-stroke depression
Inclusion criteria	Patients who had experienced stroke with aphasia; Screened positive for low mood using VAMS ‘sad’ item score >50 or Stroke Aphasic Depression Questionnaire score >6	Adults aged ≥18 years, 3 months to 5 years post-stroke, screening positive for depression (PHQ-9 score of ≥10 points or VAMS ‘sad’ item score of >50 points)	Age ≥18 years, <3 months post-stroke, subthreshold depression (CES-D ≥16, HRSD 7–17), able to attend sessions	Ischemic stroke in past 4 months, positive screen for depression, met diagnostic criteria for depression	Within 4 months of ischemic or haemorrhagic stroke, score ≥11 on GDS
Exclusion criteria	Blind, deaf, documented dementia, unable to speak English before stroke, receiving treatment for depression at time of stroke	Unable to communicate in English, receiving specialist depression treatment (except antidepressants), suicidal ideation, cognitive impairments too severe for therapy	Severe cognitive impairment, global aphasia, major medical/psychiatric conditions, taking antidepressants	Prior or current treatment for depression	Severe cognitive impairment (moved out of area, psychosis, cognitive issues, frailty)
Population	Patients who had experienced stroke with aphasia and low mood	Adults with post-stroke depression	Patients who had experienced stroke with subthreshold depression	Patients with post-stroke depression	100 stroke survivors with depression
Depression entry threshold	VAMS ‘sad’ item score >50 or Stroke Aphasic Depression Questionnaire score >6	PHQ-9 score ≥10 points or VAMS ‘sad’ item score >50 points	CES-D ≥16, HRSD 7–17	Positive screen (GDS ≥11) and met diagnostic criteria	≥11 on GDS
Setting	Recruited from hospital wards, community rehabilitation, speech therapy services and stroke groups	Recruited from hospital, community services and voluntary groups	Recruited from hospital	Recruited from hospitals	Recruited from community hospitals
Where treatment was delivered	Participants’ place of residence	Participants’ homes	Hospital	Not reported	Participants’ homes or study offices
How participants were recruited	Identified patients who had experienced stroke with aphasia and screened for low mood. Those meeting criteria were invited to participate	Screening hospital databases, stroke wards, community services and groups	Screening hospital patients who had experienced stroke	Screening hospital patients who had experienced stroke	Screening consecutive stroke in-patients from six hospitals
Were suicidal people excluded?	Not reported	Not specified	Not reported	Not reported	Not reported
Behavioural activation delivered by	Assistant psychologist supervised by clinical psychologist	Assistant psychologists or psychological well-being practitioner	Psychologist (professor specialising in psychological therapy)	Study interventionist nurses	Psychosocial nurse practitioners
Recruitment rate	105/511 (21%) screened eligible and agreed to participate	49/756 (6.5%) screened and agreed to participate	70/274 (26%) screened	101/289 screened eligible (35%)	100/416 (24%) screened and agreed to participate
Number of behavioural activation sessions	Up to 20 sessions over 3 months	Up to 15 sessions, mean 8.1 (s.d. 3.4) over 4 months	Six weekly sessions	Nine sessions over 8 weeks	Six sessions
Frequency of sessions	Weekly	Weekly	Weekly	Weekly	Weekly
Duration of behavioural activation	3 months	4 months	6 weeks	8 weeks	6 weeks
Duration of average session	57 min	58 min	50 min	Not reported	Telephone: mean 26 min; in person: mean 38 min
Number who completed behavioural activation	44/51 (86%)	20	33	44	37 (telephone) 35 (in person)
Number who completed control	Not reported	23	32	48	28
Control condition	Usual care	Usual care	Usual care	Usual care plus antidepressants	Usual care
Randomisation method	Computer-generated pseudorandom allocation sequence, stratified by site and hospital/community recruitment	Computer-generated pseudorandom allocation	Random number table	Computerised adaptive randomisation	Minimisation method
Randomised	Behavioural activation: 51; control: 54	Behavioural activation: 26; control: 23	Behavioural activation: 35; control: 35	Behavioural activation: 48; control: 53	Telephone behavioural activation: 37; in-person behavioural activation: 35; usual care: 28
Included in analysis	Behavioural activation: 51; control: 54	Behavioural activation: 18; control: 21	Behavioural activation: 33; control: 32	Behavioural activation: 44; control: 48	Telephone behavioural activation: 37; in-person behavioural activation: 35; usual care: 28
Analysis method	Intention to treat	Intention to treat	Intention to treat	Intention to treat	Per protocol
Sample size calculation	Target of 76 per group (90% power, 5% significance)	Not reported, feasibility trial	Not reported, feasibility trial	Target *N* = 101 (adequate to detect effect size of 0.5 s.d.)	75 in each of the three arms, providing a power of 92% to detect an odds ratio of 2.7 between either intervention and control or between the two intervention groups
Trial registration	Yes (ISRCTN56078830)	Yes (ISRCTN12715175)	Yes (ChiCTR2200057721)	Yes (NCT00194454)	Yes (NCT01133106)
Primary depression measure	Stroke Aphasic Depression Questionnaire	PHQ-9	CES-D, HRSD-17	HRSD	HRSD
Baseline depression score, mean (s.d.)	Behavioural activation: 11.2 (5.8); control: 9.5 (4.4)	Behavioural activation: 16.3 (4.7); control: 17.3 (4.8)	Behavioural activation: CES-D 23.69 (1.87), HRSD 13.12 (2.3); control: CES-D 24.46 (1.93), HRSD 12.38 (2.01)	Behavioural activation: 20.0 (4.5); control: 19.8 (4.2)	Telephone behavioural activation: 18.0 (3.1); in-person behavioural activation 19.1 (3.2); usual care: 18.3 (2.9)
Post-treatment assessment	3 months	6 months	6 weeks	9 weeks (follow-up, 21 weeks, 12 months, 24 month)	8 weeks post-treatment
Post-treatment depression score, mean (s.d.)	Behavioural activation: 16.9 (10.2); control: 19.2 (9.6)	Behavioural activation: 10.1 (6.9); control 14.4 (5.1)	Behavioural activation: CES-D 19.39 (1.98), HRSD 10.00 (1.67); control: CES-D 22.13 (1.18), HRSD 11.15 (1.66)	Behavioural activation: −9.8 (4.9); control: −3.6 (5.6)	Not reported
Final assessment	6 months	6 months	3 months	24 months	12 months
Final depression score, mean (s.d.)	Behavioural activation: 17.4 (10.0); control 21.9 (9.5)	Behavioural activation: 10.1 (6.9); control: 14.4 (5.1)	Behavioural activation: CES-D 16.52 (2.01), HRSD 7.54 (3.08); control: CES-D 19.16 (1.16), HRSD 9.34 (2.37)	Behavioural activation: −11.3 (6.5); control −9.3 (4.7)	Not reported
Reported harms	Not reported	Yes	No adverse events	No	None related to intervention
Ethics	Approved by Nottingham Research Ethics Committee	Approved by NHS Research Ethics Committee	Approved by hospital ethics committee	Approved by institutional review board	Approved by University of Washington Institutional Review Board

RCT, randomised controlled trial; VAMS, Visual Analog Mood Scales; PHQ-9, Patient Health Questionnaire-9; CES-D, Center for Epidemiologic Studies Depression Scale; GDS, Geriatric Depression Scale; HRSD, Hamilton Rating Scale for Depression.

#### Setting

Of the five studies, two studies were conducted in the USA, two in the UK and one in China. In all studies, participants were recruited from hospital settings (including community services and voluntary groups).

### Participants

Most studies recruited adult patients who had experienced stroke within 3–6 months after stroke, although one study (Thomas et al^28^) recruited participants between 3 months and 5 years post-stroke. Participants must have screened positive for depression, with different depression screening tools used across studies, but most required a score indicating at least mild depression. Participants with severe cognitive impairments, global aphasia or suicidal ideation were excluded.

### Intervention

In all studies, behavioural activation was the main component of the intervention, and consisted of six to 20 sessions delivered over 6 weeks to 4 months. Sessions ranged from 50 to 58 min, and were delivered in participants’ homes or at the hospital. The interventions were delivered by assistant psychologists, clinical psychologists, psychosocial nurses or nurse practitioners who were trained to deliver behavioural activation. In four of the five studies, treatment as usual (usual care) was the comparator (control condition), whereas the Mitchell et al^30^ study used usual care plus antidepressants as the control condition. Across the studies, the control conditions did not include any specific psychological interventions such as CBT.

### Outcomes

In the three of the five studies, the Hamilton Rating Scale for Depression (HRSD) was the primary depression measure.^26,29,30^ The HRSD is a 17-item clinician-administered scale assessing depression severity. The remaining two studies used a different self-report scale – the Stroke Aphasic Depression Questionnaire^27^ and the PHQ-9^28^ – as measure of primary depression. Moreover, one study^29^ used both the Center for Epidemiologic Studies Depression Scale self-report and the HRSD.

### Risk-of-bias assessment in the included studies

**Fig. 2:**
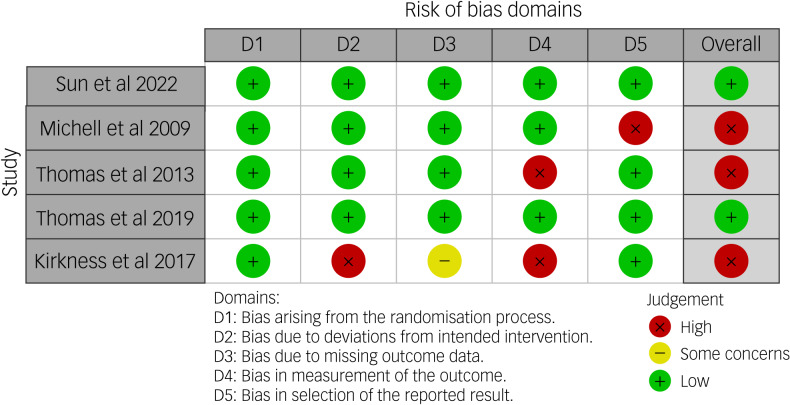
Risk-of-bias summary: review authors’ judgements about each risk-of-bias item for each included study.

**Fig. 3:**
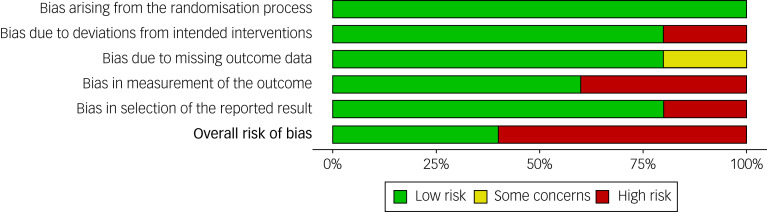
Risk-of-bias graph: review authors’ judgements about each risk-of-bias item, presented as percentages across all included studies.

Of the five studies included in the review, three studies (60%)^26,27,30^ were rated as having high risk of bias, and the remaining two studies (40%)^28,29^ were rated as having a low risk of bias in the overall assessment of risk of bias (see Figs 2 and 3). Figure 2 shows the risk-of-bias assessment for five domains across the five included studies. For the domain of bias in the selection of the reported results, the Michell et al^30^ study was rated as having ‘high risk’. Regarding bias arising from the randomisation process, all studies were rated as having ‘low risk’, because the study did provide sufficient details about the randomisation methods used. For missing outcome data, Kirkness et al^26^ was rated as ‘some concerns’, because it had high attrition.

Figure 3 shows that across all bias domains, about 60% of the studies were rated as ‘high risk’ and about 40% were rated as ‘low risk’. This reflects issues primarily related to deviations from the intended interventions, handling of missing data, measurement of outcome data and selective reporting of results.

### Effectiveness of behavioural activation versus control conditions on reducing post-stroke depression

**Fig. 4:**
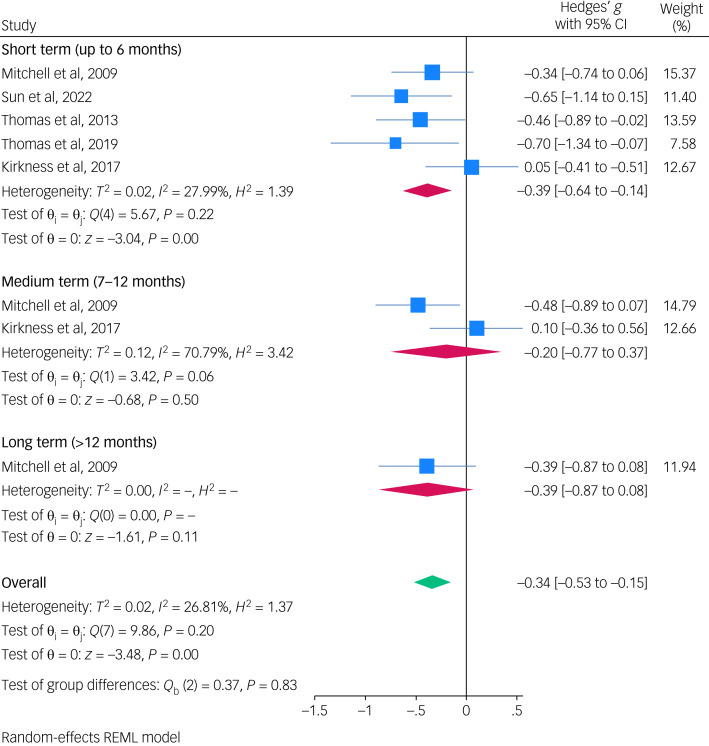
Meta-analysis showing the association between behavioural activation and post-stroke depression. REML, restricted maximum likelihood.

Figure 4 shows the short-term treatment efficacy of behavioural activation (compared with treatment as usual or usual care plus antidepressants) was associated with a decrease in depression symptoms in individuals with stroke by a mean of 0.39 s.d. (Hedges’ *g* −0.39; 95% CI −0.64 to −0.14).

Of the five studies, two studies (Mitchell et al^30^ and Kirkness et al^26^) examined medium-term treatment efficacy of behavioural activation compared with control conditions, and only one study (Mitchell et al^30^) examined long-term treatment efficacy. For medium-term efficacy, behavioural activation showed a Hedges’ *g* of −0.20 (95% CI −0. 77 to 0.37) compared with treatment as usual (see Fig. 4).

### Reporting of adverse events and harms

Out of the five included studies, one (Thomas et al^28^) provided data on adverse events occurring during the trial. Thomas and colleagues reported on both serious adverse events, defined as those requiring hospital admission or emergent care, as well as general adverse events. They documented three serious adverse events, including hospital admissions for a suicide attempt, heart attack and hernia surgery, experienced by three separate participants. Importantly, none of these major adverse events were judged to be related to the study intervention. Regarding minor adverse events, a total of 13 events were reported in ten participants overall. These included suicidal ideation, worsening health status, falls and new medical conditions emerging during the study period. When examined by study group, five adverse events occurred in four participants assigned to the intervention arm, whereas eight events were documented in six control arm participants.

## Discussion

### Key findings and interpretation

This systematic review and meta-analysis represent the first synthesis of evidence from RCTs examining the efficacy of behavioural activation compared with treatment as usual or usual care plus antidepressants for post-stroke depression. We specifically focused on analysing the effectiveness of behavioural activation versus control conditions on depression symptom improvement at multiple time points post-treatment, including short-term, medium-term and long-term follow-up. Our meta-analysis found that behavioural activation has some short-term efficacy in reducing depression symptoms compared with control conditions in individuals with post-stroke depression (Hedges’ *g* −0.39; 95% CI −0.64 to −0.14). These pooled results showed that behavioural activation treatment led to a small reduction in depression levels from baseline to short-term follow-up (up to 6 months post-treatment). This may indicate that behavioural activation is more effective than treatment as usual in improving post-stroke depressive symptoms in the short term after completing treatment. However, it is important to note that the included studies overall had a high risk of bias, which may affect the reliability of this finding. This finding should be interpreted with caution, given the methodological weaknesses of the evidence base. The durability of behavioural activation's effects is less clear at longer time points, since only two studies^26,30^ examined medium-term (7–12 months) follow-up and only one study^30^ examined long-term (>12 months) follow-up.

### Comparison with previous findings

There are systematic reviews and meta-analyses investigating the impact of behavioural activation on depression in adults with non-communicable diseases,^31^ in the adults with general depression^18,32^ and specifically in postnatal depression.^33^ Nevertheless, no systematic review or meta-analysis has explored the effectiveness of behavioural activation in addressing post-stroke depression. Our study filled this research gap by providing an examination of the efficacy of behavioural activation in the context of post-stroke depression. We found that behavioural activation had a moderate effect in improving depressive symptoms in individuals with post-stroke depression. These findings provide the first contribution to the ongoing scientific discourse on the effect of behavioural activation on post-stroke depression.

Previous systematic reviews and meta-analyses regarding psychological interventions for post-stroke depression have focused on CBT. For example, a 2018 meta-analysis involving 23 RCTs with 1972 participants by Wang et al^10^ found that CBT was associated with improved depressive symptoms compared with control groups in patients who had experienced stroke (standardised mean difference −0.83; 95% CI −1.05 to −0.60). Moreover, a 2022 systematic review and meta-analysis by Ahrens et al,^34^ involving ten studies with 672 participants, demonstrated that CBT showed large reductions in depressive symptoms (standardised mean difference 0.95; 95% CI 0.52 to 1.37). Although these effects could be somewhat overestimated because of high risk of bias and small study effects (i.e. publication bias, outcome reporting bias and clinical heterogeneity), these meta-analyses provided consistent evidence that CBT may be more effective treatment for post-stroke depression when compared with control conditions. However, as specialised expertise is required to deliver CBT, this poses challenges for implementation in underserved areas lacking mental health professionals.

The present study, however, examined a different psychological approach – behavioural activation – which could be easily delivered by non-specialists after 3 days of training. Our finding that behavioural activation reduced depressive symptoms in post-stroke patients has important practical implications for expanding access to effective mental health interventions in rural and remote settings with limited specialty care. The simplicity and effectiveness of behavioural activation implemented by lay health workers could help address the excess burden of post-stroke depression in underserved populations worldwide.

The behavioural activation therapy examined in this review was delivered by assistant psychologists, psychologists and nurses. As there were only five studies, we could not perform subgroup analyses based on the background of the clinician delivering the intervention. Therefore, it is difficult to determine if the effects of behavioural activation on post-stroke depression are influenced by the clinician's background or other factors related to the intervention itself. Future research should evaluate how the clinician's background affects primary outcomes. Understanding how clinician background affects outcomes could help determine if behavioural activation could be made more widely accessible through training specific healthcare professionals, such as nurses. This would significantly affect clinical practice by making behavioural activation more accessible, assuming appropriate training is provided.

In this review, only one study (Thomas et al^28^) reported adverse events during the trial, including both serious events requiring hospital admission and general adverse events. Thus, the limited data makes it challenging to fully evaluate the extent to which behavioural activation interventions may lead to adverse effects in the study population. In contrast, a 2023 systematic review and meta-analysis^34^ examining the effects of CBT on post-stroke depression found that none of the included studies documented any adverse effects. However, another study^35^ investigating unwanted events and side-effects in 100 patients undergoing CBT found that therapists reported approximately 372 unwanted events across 98 patients. The most common issues were negative well-being/distress (in 27% of patients) and worsening of existing symptoms (in 9% of patients). This highlights the importance of thoroughly investigating potential adverse effects associated with any psychotherapeutic interventions, including CBT and behavioural activation.

### Limitations of the review

There are several limitations to this review on behavioural activation for post-stroke depression. First, the overall sample size across studies was relatively small, leading to subanalyses that relied on a limited number of studies. The small sample sizes pose challenges in definitively establishing the effects of behavioural activation on post-stroke depression. Second, the majority of studies exhibited poor quality, indicating a potential high risk of bias in the outcomes. The difficulty in achieving blinding in psychological trials raises concerns about the introduction of placebo effects. Third, this current review focused on a single primary outcome: improvements in depressive symptoms in individuals with post-stroke depression. The review could have included studies examining other outcome measures, such as quality of life or the experiences of stroke survivors receiving behavioural activation in treating post-stroke depression. Fourth, we restricted our review to English language journals and did not examine the grey literature. Hence, relevant studies in this area may have been overlooked. Fifth, most studies excluded participants with communication difficulties; however, aphasia is common after stroke, and patients who experience aphasia have a high rate of depression.^36^ Thus, this review may miss data from a subset of the post-stroke population. Future studies should examine language-modified behavioural activation for this population. Finally, most studies did not provide long-term follow-up data. It is suggested that future research examine the long-term effectiveness of behavioural activation for post-stroke depression, including 1-year follow-ups.

### Future research

Future systematic reviews and meta-analyses examining the efficacy of behavioural activation for post-stroke depression should make efforts to include a large number of RCTs. This will allow for more definitive conclusions about the effectiveness of behavioural activation for treating post-stroke depression. The methodological quality and risk of bias of included studies needs careful assessment. Subgroup analyses should be conducted for higher-quality studies, whereas sensitivity analyses can help determine the influence of lower quality studies on outcomes. A prior study^16^ has found behavioural activation to be as effective as CBT for improving depressive symptoms and stopping depression progression in the general adult population. However, there has been limited specific investigation comparing behavioural activation and CBT for post-stroke depression. Thus, head-to-head comparisons of behavioural activation versus CBT would provide useful information on the relative efficacy of these interventions for post-stroke depression. Finally, examining how clinician background and training affects behavioural activation outcomes could highlight important implementation factors to consider.

In conclusion, evidence from this review was too little to confirm the effectiveness of behavioural activation as a useful treatment for post-stroke depression when compared with control conditions. Further high-quality studies are needed to conclusively confirm the efficacy of behavioural activation as a treatment option for post-stroke depression.

## Supporting information

Yisma et al. supplementary material 1 Yisma et al. supplementary material

Yisma et al. supplementary material 2 Yisma et al. supplementary material

Yisma et al. supplementary material 3 Yisma et al. supplementary material

## Data Availability

The data that support the findings of this study are available on request from the corresponding author, E.Y.
