# Supplementary material for: Effect of behavioural activation for individuals with post-stroke depression: systematic review and meta-analysis
Source: BJPsych Open. 2024 Jul 30;10(5):e134. doi: 10.1192/bjo.2024.721 (PMC11698145; doi:10.1192/bjo.2024.721)
Supplement: Yisma et al. supplementary material 1 — Yisma et al. supplementary material [file S205647242400721Xsup001.docx]

**Appendix 1: Search Strategy for “Effect of behavioural activation for individuals with post-stroke depression: a systematic review and meta-analysis”**

**Table S1. MEDLINE**

| **#** | **Query** |
| --- | --- |
| 1 | behavio* activat*.ti,ab,kf. |
| 2 | (behavio* adj3 (reinforce* or re-inforce*)).ti,ab,kf. |
| 3 | (behavio* adj2 (contracting or modif*)).ti,ab,kf. |
| 4 | reinforc*.ti,kf. or ((positive adj1 reinforc*) or (reinforc* adj3 (environment* or experience*))).ti,ab,kf. |
| 5 | (activit* adj2 schedul*).ti,ab,kf. |
| 6 | ((pleas* or enjoyable or rewarding) adj3 (activit* or event?)).ti,ab,kf. |
| 7 | ((operant or instrumental) adj (conditioning or learning)).ti,ab,kf. |
| 8 | (positive interaction* or avoida* coping or environmental contingenc* or contingency management).ti,ab,kf. |
| 9 | functional analysis.ti,ab,kf. |
| 10 | behavio*.mp. and (self adj (evaluat* or monitor*)).ti,ab,kf. |
| 11 | (behavio* adj (counsel* or intervention* or treatment* or therap* or psychotherap*)).ti,ab,kf. |
| 12 | (mood adj3 monitor*).ti,ab,kf. |
| 13 | Behavior Therapy/ |
| 14 | or/1-13 |
| 15 | exp stroke/ |
| 16 | brain ischemia/ and stroke.ti,ab. |
| 17 | Ischemic attack, transient/ |
| 18 | cerebral hemorrhage/ and stroke.ti,ab. |
| 19 | carotid stenosis/ and stroke.ti,ab. |
| 20 | (acute isch?emic and stroke).ti,ab. |
| 21 | (Stroke or poststroke or post stroke).ti,ab,kf. |
| 22 | (Brain Infarct* or Brain Stem Infarct*).ti,ab,kf. |
| 23 | (Lateral Medullary Syndrome* or Cerebral Infarct*).ti,ab,kf. |
| 24 | (Multi-infarct Dementia or Cerebral Artery infarct*).ti,ab,kf. |
| 25 | (transient Isch?emic attack* or cerebral isch?emi*).ti,ab,kf. |
| 26 | (cerebrovascular event* or cerebrovascular accident*).ti,ab,kf. |
| 27 | (medullary infarct* or cerebral artery occlusion or mcao).ti,ab,kf. |
| 28 | or/15-27 |
| 29 | depression/ |
| 30 | mood disorders/ |
| 31 | depressive disorder/ |
| 32 | depressive disorder, major/ |
| 33 | depressive disorder, treatment-resistant/ |
| 34 | dysthymic disorder/ |
| 35 | cyclothymic disorder/ |
| 36 | (depress* or dysthymi* or cyclothymi*).ti,ab,kf. |
| 37 | (mood or low mood or mental health or mood disorder*).ti,ab,kf. |
| 38 | or/29-37 |
| 39 | 14 and 28 and 38 |

**Table S2.** Embase

| **#** | **Query** |
| --- | --- |
| 1 | behavio* activat*.ti,ab,kw. |
| 2 | (behavio* adj3 (reinforce* or re-inforce*)).ti,ab,kw. |
| 3 | (behavio* adj2 (contracting or modif*)).ti,ab,kw. |
| 4 | reinforc*.ti,kw. or ((positive adj1 reinforc*) or (reinforc* adj3 (environment* or experience*))).ti,ab,kw. |
| 5 | (activit* adj2 schedul*).ti,ab,kw. |
| 6 | ((pleas* or enjoyable or rewarding) adj3 (activit* or event?)).ti,ab,kw. |
| 7 | ((operant or instrumental) adj (conditioning or learning)).ti,ab,kw. |
| 8 | (positive interaction* or avoida* coping or environmental contingenc* or contingency management).ti,ab,kw. |
| 9 | functional analysis.ti,ab,kw. |
| 10 | behavio*.mp. and (self adj (evaluat* or monitor*)).ti,ab,kw. |
| 11 | (behavio* adj (counsel* or intervention* or treatment* or therap* or psychotherap*)).ti,ab,kw. |
| 12 | (mood adj3 monitor*).ti,ab,kw. |
| 13 | Behavior Therapy/ |
| 14 | or/1-13 |
| 15 | cerebrovascular accident/ |
| 16 | brain ischemia/ and stroke.ti,ab. |
| 17 | Ischemic attack, transient/ |
| 18 | brain hemorrhage/ and stroke.ti,ab. |
| 19 | carotid artery obstruction/ and stroke.ti,ab. |
| 20 | (acute isch?emic and stroke).ti,ab. |
| 21 | (Stroke or poststroke or post stroke).ti,ab,kw. |
| 22 | (Brain Infarct* or Brain Stem Infarct*).ti,ab,kw. |
| 23 | (Lateral Medullary Syndrome* or Cerebral Infarct*).ti,ab,kw. |
| 24 | (Multi-infarct Dementia or Cerebral Artery infarct*).ti,ab,kw. |
| 25 | (transient Isch?emic attack* or cerebral isch?emi*).ti,ab,kw. |
| 26 | (cerebrovascular event* or cerebrovascular accident*).ti,ab,kw. |
| 27 | (medullary infarct* or cerebral artery occlusion or mcao).ti,ab,kw. |
| 28 | or/15-27 |
| 29 | depression/ |
| 30 | mood disorder/ |
| 31 | chronic depression/ or post-stroke depression/ or recurrent brief depression/ or seasonal affective disorder/ |
| 32 | major depression/ |
| 33 | treatment resistant depression/ |
| 34 | dysthymia/ |
| 35 | cyclothymia/ |
| 36 | (depress* or dysthymi* or cyclothymi*).ti,ab,kw. |
| 37 | (low mood or mood disorder* or affective disorder*).ti,ab,kw. |
| 38 | or/29-37 |
| 39 | 14 and 28 and 38 |

**Table S3.** Ovid Emcare

| **#** | **Query** |
| --- | --- |
| 1 | behavio* activat*.ti,ab,kw. |
| 2 | (behavio* adj3 (reinforce* or re-inforce*)).ti,ab,kw. |
| 3 | (behavio* adj2 (contracting or modif*)).ti,ab,kw. |
| 4 | reinforc*.ti,kw. or ((positive adj1 reinforc*) or (reinforc* adj3 (environment* or experience*))).ti,ab,kw. |
| 5 | (activit* adj2 schedul*).ti,ab,kw. |
| 6 | ((pleas* or enjoyable or rewarding) adj3 (activit* or event?)).ti,ab,kw. |
| 7 | ((operant or instrumental) adj (conditioning or learning)).ti,ab,kw. |
| 8 | (positive interaction* or avoida* coping or environmental contingenc* or contingency management).ti,ab,kw. |
| 9 | functional analysis.ti,ab,kw. |
| 10 | behavio*.mp. and (self adj (evaluat* or monitor*)).ti,ab,kw. |
| 11 | (behavio* adj (counsel* or intervention* or treatment* or therap* or psychotherap*)).ti,ab,kw. |
| 12 | (mood adj3 monitor*).ti,ab,kw. |
| 13 | Behavior Therapy/ |
| 14 | or/1-13 |
| 15 | cerebrovascular accident/ |
| 16 | brain ischemia/ and stroke.ti,ab. |
| 17 | transient ischemic attack/ |
| 18 | brain hemorrhage/ and stroke.ti,ab. |
| 19 | carotid artery obstruction/ and stroke.ti,ab. |
| 20 | (acute isch?emic and stroke).ti,ab. |
| 21 | (Stroke or poststroke or post stroke).ti,ab,kw. |
| 22 | (Brain Infarct* or Brain Stem Infarct*).ti,ab,kw. |
| 23 | (Lateral Medullary Syndrome* or Cerebral Infarct*).ti,ab,kw. |
| 24 | (Multi-infarct Dementia or Cerebral Artery infarct*).ti,ab,kw. |
| 25 | (transient Isch?emic attack* or cerebral isch?emi*).ti,ab,kw. |
| 26 | (cerebrovascular event* or cerebrovascular accident*).ti,ab,kw. |
| 27 | (medullary infarct* or cerebral artery occlusion or mcao).ti,ab,kw. |
| 28 | or/15-27 |
| 29 | depression/ |
| 30 | mood disorders/ |
| 31 | chronic depression/ or post-stroke depression/ or recurrent brief depression/ or seasonal affective disorder/ |
| 32 | major depression/ |
| 33 | treatment resistant depression/ |
| 34 | dysthymia/ |
| 35 | cyclothymia/ |
| 36 | (depress* or dysthymi* or cyclothymi*).ti,ab,kw. |
| 37 | (low mood or mood disorder* or affective disorder*).ti,ab,kw. |
| 38 | or/29-37 |
| 39 | 14 and 28 and 38 |

**Table S4.** APA PsycInfo

| **#** | **Query** |
| --- | --- |
| 1 | behavior activation system/ |
| 2 | behavio* activat*.tw. |
| 3 | (behavio* adj3 (reinforce* or re-inforce*)).tw. |
| 4 | exp reinforcement/ |
| 5 | (behavio* adj2 (contracting or modif*)).tw. |
| 6 | (reinforc* or ((positive adj1 reinforc*) or (reinforc* adj3 (environment* or experience*)))).tw. |
| 7 | (activit* adj2 schedul*).tw. |
| 8 | Planned Behavior/ |
| 9 | ((pleas* or enjoyable or rewarding) adj3 (activit* or event?)).tw. |
| 10 | ((operant or instrumental) adj (conditioning or learning)).tw. |
| 11 | exp operant conditioning/ |
| 12 | (positive interaction* or avoida* coping or environmental contingenc* or contingency management).tw. |
| 13 | exp contingency management/ |
| 14 | functional analysis.tw. |
| 15 | behavio*.mp. and (self adj (evaluat* or monitor*)).tw. |
| 16 | self-management/ and behavior change/ |
| 17 | (behavio* adj (counsel* or intervention* or treatment* or therap* or psychotherap*)).tw. |
| 18 | (mood adj3 monitor*).tw. |
| 19 | Behavior Therapy/ |
| 20 | or/1-19 |
| 21 | cerebrovascular accidents/ |
| 22 | cerebral ischemia/ and stroke.ti,ab. |
| 23 | cerebral hemorrhage/ and stroke.ti,ab. |
| 24 | (acute isch?emic and stroke).ti,ab. |
| 25 | (stroke or poststroke or post stroke).tw. |
| 26 | (Brain Infarct* or Brain Stem Infarct*).tw. |
| 27 | (Lateral Medullary Syndrome* or Cerebral Infarct*).tw. |
| 28 | (Multi-infarct Dementia or Cerebral Artery infarct*).tw. |
| 29 | (transient Isch?emic attack* or cerebral isch?emi*).tw. |
| 30 | (cerebrovascular event* or cerebrovascular accident*).tw. |
| 31 | (medullary infarct* or cerebral artery occlusion or mcao).tw. |
| 32 | or/21-31 |
| 33 | Atypical Depression/ or Reactive Depression/ or Recurrent Depression/ or Late Life Depression/ |
| 34 | mood disorders/ |
| 35 | affective disorders/ |
| 36 | Major Depression/ |
| 37 | Treatment Resistant Depression/ |
| 38 | dysthymic disorder/ |
| 39 | cyclothymic disorder/ |
| 40 | (depress* or dysthymi* or cyclothymi*).tw. |
| 41 | (low mood or mood disorder* or affective disorder*).tw. |
| 42 | or/33-41 |
| 43 | 20 and 32 and 42 |

**Table S5. Cochrane Library**

|  | **Query** |
| --- | --- |
| #1 | behavio* next activat* ti,ab, kw |
| #2 | (behavio* Near/3 (reinforce* or re-inforce*)) ti,ab,kw |
| #3 | (behavio* near/2 (contracting or modif*)) ti,ab,kw |
| #4 | reinforc* or ((positive near/1 reinforc*) or (reinforc* near/3 (environment* or experience*))):ti,ab, kw |
| #5 | (activit* near/2 schedul*) ti,ab,kw. |
| #6 | ((pleas* or enjoyable or rewarding) near/3 (activit* or event?)) ti,ab,kw |
| #7 | ((operant or instrumental) near (conditioning or learning))ti,ab,kw. |
| #8 | (positive next interaction* or avoida* next coping or environmental next contingenc* or contingency next management) ti,ab,kw. |
| #9 | "functional analysis" ti,ab,kw. |
| #10 | behavio* and (self near (evaluat* or monitor*)) ti,ab,kw. |
| #11 | (behavio* near (counsel* or intervention* or treatment* or therap* or psychotherap*)) ti,ab,kw |
| #12 | (mood near/3 monitor*) ti,ab,kw. |
| #13 | MeSH descriptor: [Behavior Therapy] this term only |
| #14 | #1 or #2 or #3 or #4 or #5 or #6 or #7 or #8 or #9 or #10 or #11 or #12 # or #13 |
| #15 | MeSH descriptor: [Stroke] explode all trees |
| #16 | (Stroke or poststroke or "post stroke") ti,ab,kw |
| #17 | (Brain next Infarct* or Brain next Stem next Infarct*) ti,ab,kw. |
| #18 | (Lateral next Medullary next Syndrome* or Cerebral next Infarct*) ti,ab,kw. |
| #19 | ("Multi-infarct Dementia" or Cerebral next Artery next infarct*) ti,ab,kw. |
| #20 | (cerebrovascular next event* or cerebrovascular next accident*) ti,ab,kw. |
| #21 | (medullary next infarct* or "cerebral artery occlusion" or mcao) ti,ab,kw. |
| #22 | #15 or #16 or #17 or #18 or #19 or #20 or #21 |
| #23 | MeSH descriptor: [Depression] this term only |
| #24 | MeSH descriptor: [Mood Disorders] this term only |
| #25 | MeSH descriptor: [Depressive Disorder] this term only |
| #26 | MeSH descriptor: [Depressive Disorder, Major] this term only |
| #27 | MeSH descriptor: [Depressive Disorder, Treatment-Resistant] this term only |
| #28 | MeSH descriptor: [Dysthymic Disorder] this term only |
| #29 | MeSH descriptor: [Cyclothymic Disorder] this term only |
| #30 | (depress* or dysthymi* or cyclothymi*) ti,ab,kw. |
| #31 | ("low mood" or mood next disorder* or affective next disorder*) ti,ab,kw. |
| #32 | #23 or #24 or #25 or #26 or #27 or #28 or #29 or #30 or #31 |
| #33 | #14 and #22 and #32 |
