# Supplementary material for: Effect of behavioural activation for individuals with post-stroke depression: systematic review and meta-analysis
Source: BJPsych Open. 2024 Jul 30;10(5):e134. doi: 10.1192/bjo.2024.721 (PMC11698145; doi:10.1192/bjo.2024.721)
Supplement: Yisma et al. supplementary material 3 — Yisma et al. supplementary material [file S205647242400721Xsup003.docx]

**Appendix 3. Characteristics of excluded studies**

| **Study** | **Reason for exclusion** |
| --- | --- |
| [Chang 2011](https://www.tandfonline.com/doi/abs/10.1310/tsr1805-525) | Wrong intervention |
| [Thomas 2013](https://journals.sagepub.com/doi/10.1177/0269215513489579) | Wrong outcome |
| [Fang 2017](https://www.ncbi.nlm.nih.gov/pmc/articles/PMC5654182/) | Wrong intervention |
| [Mitchell 2008](https://www.strokejournal.org/article/S1052-3057(07)00206-6/fulltext) | Wrong study design |
| [Lenzo 2019](https://old.jpsychopathol.it/wp-content/uploads/2019/07/03_Lenzo-1.pdf) | Wrong intervention |
| [Bhardwaj 2018](https://journals.lww.com/annalsofian/fulltext/2018/21030/efficacy_of_cranial_electrical_stimulation_and.5.aspx) | Wrong intervention |
| [Humphreys 2015](https://journals.sagepub.com/doi/10.1177/0269215514537656) | Wrong outcome |
| [Byun 2021](https://journals.lww.com/rehabnursingjournal/abstract/2021/07000/brief_psychosocial_intervention_to_address.7.aspx) | Wrong study design |
